# Unique pharmacodynamic properties and low abuse liability of the μ-opioid receptor ligand (S)-methadone

**DOI:** 10.21203/rs.3.rs-2644719/v1

**Published:** 2023-03-23

**Authors:** Michael Michaelides, Marjorie Levinstein, Paulo De Oliveira, Nil Casajuana-Martin, Cesar Quiroz, Reece Budinich, Rana Rais, William Rea, Emilya Ventriglia, Natàlia Llopart, Verònica Casadó-Anguera, Estefanía Moreno, Donna Walther, Grant Glatfelter, David Weinshenker, Carlos Zarate, Vicent Casado, Michael Baumann, Leonardo Pardo, Sergi Ferre

**Affiliations:** NIH; National Institute on Drug Abuse; National Institute on Drug Abuse; Universitat Autònoma Barcelona; National Institute on Drug Abuse; National Institute on Drug Abuse; Johns Hopkins School of Medicine; National Institute on Drug Abuse; National Institute on Drug Abuse; Universitat de Barcelona; Universitat de Barcelona; Department of Biochemistry and Molecular Biomedicine, Faculty of Biology, Institute of Biomedicine of the University of Barcelona, University of Barcelona; National Institute on Drug Abuse; National Institute on Drug Abuse; NIMH; University of Barcelona; National Institute on Drug Abuse; Universitat Autonoma de Barcelona; National Institute on Drug Abuse

**Keywords:** opioid, computational model, NMDAR

## Abstract

(R,S)-methadone ((R,S)-MTD) is a racemic μ-opioid receptor (MOR) agonist comprised of (R)-MTD and (S)-MTD enantiomers used for the treatment of opioid use disorder (OUD) and pain. (R)-MTD is used as an OUD treatment, has high MOR potency, and is believed to mediate (R,S)-MTD’s therapeutic efficacy. (S)-MTD is in clinical development as an antidepressant and is considered an N-methyl-D-aspartate receptor (NMDAR) antagonist. In opposition to this purported mechanism of action, we found that (S)-MTD does not occupy NMDARs in vivo in rats. Instead, (S)-MTD produced MOR occupancy and induced analgesia with similar efficacy as (R)-MTD. Unlike (R)-MTD, (S)-MTD was not self-administered and failed to increase locomotion or extracellular dopamine levels indicating low abuse liability. Moreover, (S)-MTD antagonized the effects of (R)-MTD in vivo and exhibited unique pharmacodynamic properties, distinct from those of (R)-MTD. Specifically, (S)-MTD acted as a MOR partial agonist with a specific loss of efficacy at the MOR-galanin 1 receptor (Gal1R) heteromer, a key mediator of the dopaminergic effects of opioids. In sum, we report novel and unique pharmacodynamic properties of (S)-MTD that are relevant to its potential mechanism of action and therapeutic use, as well as those of (R,S)-MTD.

## Introduction

Opioid medications are potent and efficacious analgesics, but their use can be associated with serious adverse effects such as tolerance, dependence, and respiratory depression. (R,S)-methadone ((R,S)-MTD) is an opioid medication used as an analgesic and a maintenance therapy for opioid use disorder (OUD) ^1,2^. (R,S)-MTD is a long-acting μ-opioid receptor (MOR) agonist that is comprised of equal amounts of (R)-MTD and (S)-MTD enantiomers. The therapeutic properties of (R,S)-MTD are believed to be mediated by the pharmacological actions of (R)-MTD^3^, which is also prescribed alone as a maintenance therapy for OUD^4^.

(S)-MTD, historically considered the inactive enantiomer of (R,S)-MTD, is now under clinical development as a treatment for depression^5–7^. Although its precise *in vivo* pharmacology is not well understood, (S)-MTD’s antidepressant mechanism of action is attributed to N-methyl-D-aspartate receptor (NMDAR) antagonism^5–9^. Specifically, (S)-MTD has ~2.6-7.4 μM affinity at NMDARs^10^ and produces behavioral and neurochemical effects in rodents that are similar to those produced by ketamine^6,7^, a known NMDAR antagonist and effective antidepressant. However, recent evidence implicates MOR agonism as a relevant mechanism for ketamine’s antidepressant effects, its abuse liability, and *in vivo* pharmacology^11–14^. Furthermore, (S)-MTD’s affinity for the MOR is ~300 times greater than its affinity for the NMDAR^10,15^. Finally, (S)-MTD is an established MOR agonist, whereas its NMDAR actions involve noncompetitive antagonism^10^.

(R,S)-MTD and its enantiomers are classified as Schedule II controlled substances by the United States Drug Enforcement Administration. Nevertheless, (R,S)-MTD produces weaker activation of midbrain dopamine systems and has lower abuse liability when compared to other opioids^16^. The reduced dopaminergic effects of (R,S)-MTD are dependent on its unique, weak interaction with MOR-galanin 1 receptor (Gal_1_R) heteromers specifically expressed in the ventral tegmental area (VTA). MOR-Gal_1_R in the VTA are known to mediate the activation of the dopaminergic system by opioids^16^, but the effects of (R) and (S) enantiomers of MTD at these heteromers are unknown.

In order to explore the analgesic and abuse liability profiles of (R,S)-MTD and its enantiomers, and to address the gaps in knowledge about these compounds, we performed an in-depth *in vitro, in vivo* and *in silico* pharmacological characterization of (R,S)-MTD, (R)-MTD and (S)-MTD. Our findings provide a mechanistic basis for the differential *in vitro* and *in vivo* properties of the enantiomers, which may impact on their clinical utility.

## Results

### (R)-MTD and (S)-MTD preferentially bind and activate MOR

**Figure 1:**
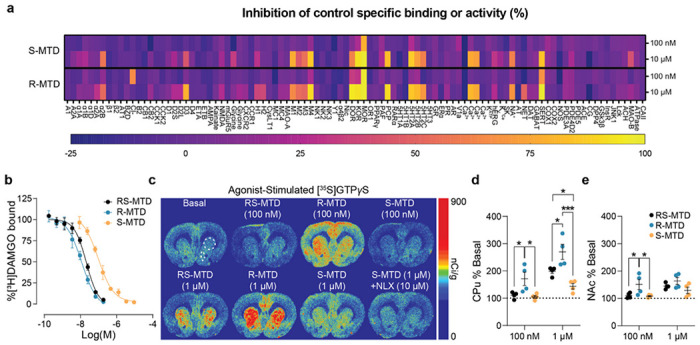
Methadone and its enantiomers are MOR agonists. **a,** Receptor and enzyme competitive screen at two concentrations (100 nM and 10 μM) of (S)- and (R)-MTD. **b,** Competition binding assays of (S)-MTD (orange), (R)-MTD (blue), or (R,S)-MTD (black) versus [^3^H]DAMGO. **c-e,** Representative slices (**c**) and analysis from methadone-stimulated [^35^S]GTP*γ*S autoradiography for CPu (**d**, upper circle) and NAc (**e**, lower ellipse). Values are shown as mean ± standard error of the mean. CPu = caudate putamen; MOR = mu opioid receptor; MTD = methadone; NLX = naloxone; NAc = nucleus accumbens. *P < 0.05, **P < 0.01, ***P < 0.001.

Each enantiomer was tested for its ability to competitively inhibit binding or activity at a panel of 98 receptors and enzymes that are known targets for drugs of abuse and CNS medications. At 10 μM, (R)-MTD inhibited binding at several receptors (Fig. 1a), while at 100 nM, (R)-MTD inhibited binding only at MOR (98%) and SERT (68%). At 10 μM, (S)-MTD inhibited binding at several receptors, while at 100 nM, (S)-MTD inhibited binding only at MOR (79%). We derived each enantiomer’s affinity at MOR using inhibition of [^3^H]DAMGO binding in rat brain tissue. The Ki values obtained were 15.6 ± 0.1 nM for (R,S)-MTD, 7.5 ± 0.1 nM for (R)-MTD and 60.5 ± 0.1 nM for (S)-MTD (Fig. 1b).

Agonist-stimulated [^35^S]GTP*γ*S autoradiography in rat brain sections was used to examine the ability of (R,S)-MTD and its enantiomers to activate MOR (Fig. 1c). At 100 nM, only (R)-MTD increased [^35^S]GTP*γ*S recruitment in the caudate putamen (CPu) (171%) and nucleus accumbens (NAc) (151%) (Fig. 1d, e). By contrast, at 1 μM all drugs increased [^35^S]GTP*γ*S recruitment in CPu (R,S: 199%; R: 270%; S: 144%) and NAc (R,S: 145%; R: 164%; S: 120%) (Fig. 1d, e). At the 1 μM concentration, (R)-MTD showed significantly greater [^35^S]GTP*γ*S recruitment compared to (R,S)-MTD (*P* = 0.01) and (S)-MTD (*P* < 0.001) in CPu. Additionally, (R,S)-MTD showed greater [^35^S]GTP*γ*S recruitment compared to (S)-MTD (*P* = 0.028). Finally, the regional distribution of (S)-MTD-induced [^35^S]GTP*γ*S recruitment was blocked by naloxone (10 μM) indicating opioid receptor involvement (Fig. 1c).

### (S)-MTD exhibits similar analgesic efficacy as (R)-MTD and (R,S)-MTD

**Figure 2:**
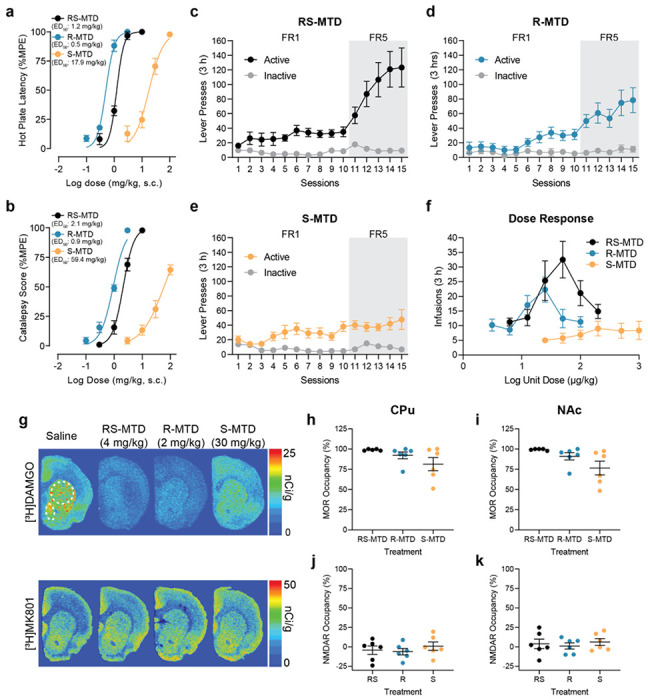
Analgesic, cataleptic and differential abuse liability profile of (R,S)-MTD, (R)-MTD, and (S)-MTD. **a-b,** Dose response curves of hotplate latency (**a**) and catalepsy (**b**) for (R,S)-MTD (black), (R)-MTD (blue) and (S)-MTD (orange), **c-e,** Lever presses during IVSA training for (R,S)-MTD (100 μg/kg/infusion, **c**), (R)-MTD (50 μg/kg/infusion, **d**), and (S)-MTD (500 μg/kg/infusion, **e**). **f,** IVSA dose responses for (R,S)-MTD, (R)-MTD, and (S)-MTD. **g-k,** Representative slices (**g**) and analysis of receptor occupancy by (R,S)-MTD, (R)-MTD, or (S)-MTD of MORs ([^3^H]DAMGO, 5nM) in CPU (**h**, upper circle) and NAc (**i**, lower ellipse) or NMDARs ([^3^H]MK-801, 5nM) (**j-k**). Values are shown as mean ± standard error of the mean. CPu = caudate putamen; ED_50_ = half maximal effective dose; FR = fixed-ratio schedule; IVSA = intravenous self-administration; MOR = μ opioid receptor; MPE = maximum possible effect; MTD = methadone; NAc = nucleus accumbens; NMDAR = N-methyl-D-aspartate receptor.

The hot plate test was used to evaluate analgesic effects in rats. (R,S)-MTD, (R)-MTD, and (S)-MTD demonstrated full agonistic activity, with ED_50_ values (%MPE, maximum possible effect) of 1.2, 0.5 and 17.9 mg/kg, respectively (Figs. 2a, **Supplementary Fig. 1**). Conversely, when evaluating catalepsy score in rats, (S)-MTD behaved as a partial agonist, unable to achieve maximal cataleptic effects, even at 100 mg/kg. The high-dose effect was ~60% of the maximal cataleptic effects of both (R)-MTD and (R,S)-MTD, which were observed at 3 and 10 mg/kg, respectively (Figs. 2b, **Supplementary Fig. 1**). The cataleptic ED_50_ values (%MPE) for (R,S)-MTD, (R)-MTD and (S)-MTD were 2.1, 0.9 and 59.4 mg/kg, respectively, which were two- to three-fold higher than their analgesic ED_50_ values. For the three drugs, the maximal cataleptic effect corresponded to the minimal dose required to produce significant hypothermia (**Supplementary Fig. 1**). (S)-MTD-induced catalepsy did not observably saturate, and toxic higher doses were not employed. Overall, these experiments demonstrate a MOR agonistic profile of (S)-MTD, with a lower potency and a possibly lower intrinsic efficacy compared to (R)-MTD. Finally, (R)-MTD and (S)-MTD did not differ in their propensity to interact with efflux transporters or in relation to CYP-dependent metabolism (**Supplementary Fig. 2**), indicating that the two enantiomers would demonstrate similar metabolic profiles *in vivo*.

### (S)-MTD exhibits lower abuse liability than (R)-MTD and (R,S)-MTD

There is evidence that (R,S)-MTD is self-administered in humans^17^ and rats^18^. However, the intravenous self-administration (IVSA) of (R)-MTD and (S)-MTD has not been reported. Moreover, depending on the dose administered, (R,S)-MTD can have either rewarding or aversive effects in rats^19^. IVSA, the standard preclinical approach for predicting abuse liability of drugs in humans^20^, was used to evaluate the reinforcing effects of (R,S)-MTD and its enantiomers in rats. First, we performed dose finding experiments to determine the dose of each drug that maintained IVSA. Rats exposed to various doses of (R)-MTD readily self-administered 50 μg/kg/infusion and consumed a maximum of ~2 mg/kg at the highest dose (**Supplementary Fig. 3**). Rats exposed to (S)-MTD never acquired IVSA, even at high unit doses, and did not show any evidence of dose response. Nevertheless, when (R)-MTD-trained rats were switched to (S)-MTD, they showed reliable IVSA at 500 μg/kg/infusion (S)-MTD. The switched rats consumed a cumulative dose of ~30 mg/kg at the highest (S)-MTD dose (**Supplementary Fig. 3**).

Next, we performed IVSA studies on another cohort of rats trained on either (R)-MTD (50 μg/kg/infusion), (R,S)-MTD (100 μg/kg/infusion), or (S)-MTD (500 μg/kg/infusion) (Fig. 2c–e). For the first 10 days of training, rats were on a fixed-ratio 1 (FR1) schedule. During this time, rats in all three groups learned to discriminate the active from inactive lever. On the 11^th^ session, the schedule was increased to FR5 (5 presses for 1 infusion). Whereas rats trained on (R)- and (R,S)-MTD adjusted lever press rates to maintain stable infusion rates, rats trained on (S)-MTD did not. We then performed a dose response assessment of IVSA (Fig. 2f). Rats trained on (R)- or (R,S)-MTD displayed the typical inverted-U shaped dose-response curve, but rats trained on (S)-MTD showed no evidence of dose response. Rats given (R)-MTD showed peak infusion rates at 25 μg/kg, while rats given (R,S)-MTD peaked at 50 μg/kg. Notably, rats trained on (R,S)-MTD had more infusions at the peak unit dose than those on (R)-MTD, and the (R,S)-MTD curve was significantly shifted to the right indicating that larger drug amounts were required to reach the same level of reinforcement.

### (R)-MTD and (S)-MTD preferentially bind to MOR **in vivo**

Based on drug exposure levels from the studies noted above, and prior reports on antidepressant-like doses of (S)-MTD used in rats^6,7^, we assessed the binding of (R,S)-MTD and its enantiomers at MOR and NMDAR *in vivo*. Rats were injected with saline (1 ml/kg, sc), (R,S)-MTD (4 mg/kg, sc), (R)-MTD (2 mg/kg, sc), or (S)-MTD (30 mg/kg, sc) 30 min before decapitation, blood collection, and brain extraction. Brains were split into two hemispheres. One hemisphere was used to assess drug amounts whereas the other was sectioned (20 μm) and subjected to autoradiography using [^3^H]DAMGO or [^3^H]MK-801 to examine occupancy at MORs or NMDARs, respectively (Fig. 2g). After 2 mg/kg of (R)-MTD, total/estimated free^21,22^ drug concentration was 640/19.2 nM ± 136/4.1 nM in plasma and 1.1/0.03 μM ± 0.15/0.005 μM in brain. After 30 mg/kg of (S)-MTD, total/free drug was 5/0.15 μM ± 0.6/0.017 μM in plasma and 11.5/0.35 μM ± 1.5/0.046 μM in brain. Finally, after 4 mg/kg of (R,S)-MTD, total/free (R)-MTD was 551/16.5 nM ± 119/3.6 nM in plasma and 1.3/0.04 μM ± 0.3/0.008 μM in brain, while total/free (S)-MTD was 580/17.4 nM ± 111/3.3 nM in plasma and 1.4/0.04 μM ± 0.3/0.009 μM in brain. The free (i.e., unbound) drug concentration provides the most accurate measure of biophase drug concentration able to engage pharmacological targets in plasma or brain^23^. Since the free concentration of (R,S)-MTD and its enantiomers is reported to be ~3% of total concentration^21,22^, it is unlikely that (R,S)-MTD or its enantiomers reach sufficient concentration to engage with NMDAR *in vivo*. By contrast, the free concentrations shown here align well with each drug’s Ki at MOR. As predicted by the free concentrations of each drug, we found that 4 mg/kg (R,S)-MTD, 2 mg/kg (R)-MTD, and 30 mg/kg (S)-MTD produced near total (99%, 91%, and 79% respectively) occupancy of MORs 30 min after injection (Fig. 2h, i). Importantly, none of the drugs produced any NMDAR occupancy (Fig. 2j, k).

### (R)-MTD and (S)-MTD do not produce MOR desensitization

Decreases in MOR density and desensitization contribute to the development of opioid tolerance^24^. In contrast to other MOR agonists, (R,S)-MTD does not produce tolerance, due to its ability to induce MOR internalization and recycling of re-sensitized MOR^25^. Thus, we examined to what extent repeated exposure to (R)-MTD (2 mg/kg, sc), (R,S)-MTD (4 mg/kg, sc), or (S)-MTD (30 mg/kg, sc) lead to changes in MOR density and G protein activation using [^3^H]DAMGO and DAMGO-stimulated [^35^S]GTP*γ*S autoradiography. We found that neither (R,S)-MTD nor its enantiomers produced any effect on MOR density or G protein activity (**Supplementary Fig. 4**).

### Divergent pharmacodynamic effects of (R)-MTD and (S)-MTD at MOR in the VTA

**Figure 3:**
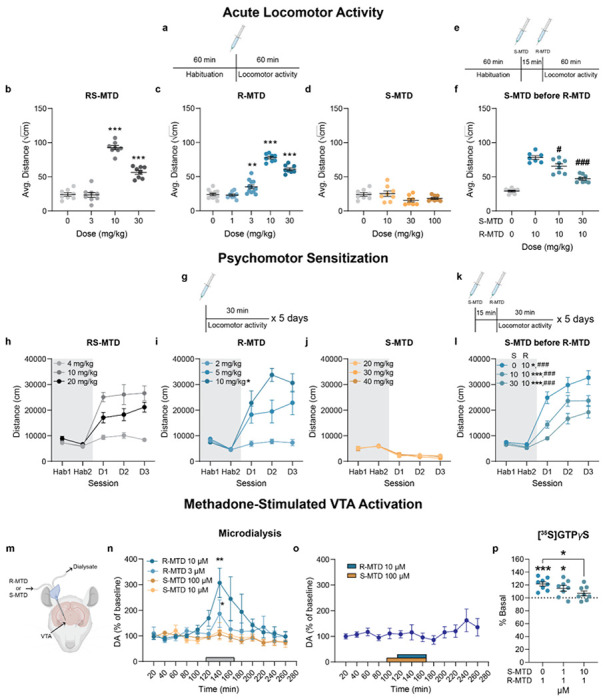
VTA-dependent neurochemical and behavioral effects of (R)-MTD and (S)-MTD. Created with BioRender.com. **a-f,** acute locomotor activation schematics (**a, e**) and analysis with (R,S)-MTD (**b**), (R)-MTD (**c**), or (S)-MTD (**d**) alone, or pretreatment of (S)-MTD before (R)-MTD (**f**). Data shown as the average of the square root of centimeters traveled per ten minutes. Asterisks are compared to saline; pound symbols are compared to (R)-MTD alone. **g-l,** psychomotor sensitization schematics (**g, k**) and analysis with (R,S)-MTD (**h**), (R)-MTD (**i**), or (S)-MTD alone (**j**), or pretreatment of (S)-MTD before (R)-MTD (**I**). Asterisks are comparison between D1 and D2; pound symbols are comparison between D1 and D3. **m-o,** effect of intracranial perfusion of (R)-MTD and (S)-MTD in the VTA on somato-dendritic dopamine release from **in vivo** microdialysis experiments. Values represent mean dopamine concentrations as a percentage of baseline ± standard error of the mean (average of 5 samples before the enantiomer administration). The rectangles in the x axis indicate the period of corresponding enantiomer perfusion. In **o**, co-perfusion of both enantiomers, with (S)-MTD (100 μM) beginning 20 min before (R)-MTD (10 μM). **p,** Analysis of [^35^S]GTP*γ*S recruitment by R-MTD (1 μM) with or without preincubation of S-MTD (1 μM or 10 μM) in the VTA. Values are shown as mean ± standard error of the mean. D1, 2, 3 = day 1, 2, or 3; Hab = habituation, MTD = methadone, VTA = ventral tegmental area. *,^#^ *P* < 0.05, **P < 0.01, ***,^###^ *p* < 0.001.

In view of the apparent lower reinforcing efficacy of (S)-MTD in rats, we next examined effects of the (R,S)-MTD and its enantiomers on locomotor activity in mice. In contrast to rats, which become cataleptic following opioid exposure, mice display dose-dependent increases in locomotion^26–30^. This opioid-induced hyperlocomotion is dependent on dopaminergic activation^31–33^, namely the activation of MORs expressed on GABA afferents onto VTAdopamine neurons^34^. Additionally, locomotor activation can distinguish between full and partial MOR agonists, with partial agonists producing graded increases dependent on efficacy^35^. We found that (R,S)-MTD and (R)-MTD increased locomotion, but (S)-MTD did not (Fig. 3a–d). Specifically, after 60 minutes of habituation, (R,S)-MTD produced a significant locomotor activation at 10 mg/kg (sc) but not at 3 mg/kg (sc), and 30 mg/kg (sc) was less effective than 10 mg/kg. An inverted U shape effect was also observed with (R)-MTD, which was more potent and effective at 3 mg/kg (sc). (S)-MTD did not produce any significant locomotor-activating effects, even at 100 mg/kg (sc; Fig. 3d). Moreover, when administered 15 min before (R)-MTD, (S)-MTD (10, 30 mg/kg, sc) dose-dependently counteracted the locomotor-stimulating effect of (R)-MTD (10 mg/kg, sc; Fig. 3e–f).

Repeated administration of opioids in rodents leads to psychomotor sensitization, which is classically known to depend on activation of MOR localized in the VTA^36^. We habituated mice to open-field chambers for two days before giving repeated injections of (R)-MTD (2, 5, or 10 mg/kg, ip), (R,S)-MTD (4, 10, or 20 mg/kg, ip), or (S)-MTD (20, 30, or 40 mg/kg, ip) for three days (Fig. 3g–j). (R)-MTD at 5 or 10 mg/kg and (R,S)-MTD at 10 or 20 mg/kg led to significant acute locomotion each day. Only (R)-MTD produced psychomotor sensitization at 10 mg/kg (D1 vs D2: *P* = 0.01). Mice treated with (R,S)-MTD and (S)-MTD failed to show sensitization at any dose. We also investigated whether (S)-MTD pretreatment (10 or 30 mg/kg, ip) would prevent (R)-MTD-induced (10 mg/kg, ip) sensitization (Fig. 3k–l). As before, we found that (S)-MTD dose-dependently decreased acute locomotion produced by (R)-MTD, however, it did not prevent psychomotor sensitization ((S)-MTD 0 mg/kg: D1 vs D3: *P* = 0.0003; (S)-MTD 10 mg/kg D1 vs D3: *P* < 0.0001; (S)-MTD 30 mg/kg: D1 vs D3: *P* < 0.0001).

In view of the apparent lower reinforcing efficacy of (S)-MTD in rats we next examined whether the enantiomers of (R,S)-MTD could stimulate MOR receptors in the VTA which are involved in opioid reinforcement. In particular, the weak interaction of (R,S)-MTD with MOR-Gal_1_R heteromers in rat VTA is thought to underlie its reduced dopaminergic activation and lower abuse liability^16^. Thus, we studied the effects of (R)-MTD and (S)-MTD perfusion into the rat VTA, using *in vivo* microdialysis. We recently showed that the intracranial perfusion of (R,S)-MTD in the VTA was less potent and efficacious than other opioids (e.g., morphine, fentanyl and DAMGO) at eliciting somatodendritic dopamine release^16^. This reduced effect was attributed to (R,S)-MTD’s weak activation of MOR-Gal_1_R. Here, we show that local perfusion of (R)-MTD into the VTA produced a concentration-dependent increase in extracellular dopamine, with a significant increase at 3 μM and a larger increase at 10 μM (Fig. 3m, n), showing similar potency to that previously obtained with morphine^16^. In contrast, (S)-MTD did not induce any significant effect on extracellular dopamine levels when perfused up to 100 μM (Fig. 3n). Notably, the 100 μM concentration of (S)-MTD completely counteracted the effect of (R)-MTD on dopamine release (Fig. 3o).

In rat brain slices containing the VTA, 1 μM (R)-MTD significantly increased [^35^S]GTP*γ*S recruitment (121.6%, *P* = 0.0003), which was prevented by preincubation with (S)-MTD. As shown in Fig. 3p, 1 μM (S)-MTD + 1 μM (R)-MTD significantly increased [^35^S]GTPγS recruitment (115%, *P* = 0.012), while 10 μM (S)-MTD + 1 μM (R)-MTD (107%) did not. The ability of (S)-MTD (10 μM) to reduce [^35^S]GTP*γ*S recruitment by (R)-MTD (1 μM) was significant (*P* = 0.039). The [^35^S]GTP*γ*S results also demonstrate a qualitatively different profile for (R)-MTD and (S)-MTD when comparing effects across different brain areas, with (S)-MTD showing a detectable efficacy in the striatum (Fig. 1d, e) and significantly counteracting the effect of R-MTD in the VTA, but not in the CPu or NAc (**Supplementary Fig. 5**).

### Divergent pharmacodynamic effects of (R)-MTD and (S)-MTD at the MOR-Gal_1_R heteromer

We next investigated the possibility of divergent pharmacodynamic effects MTD enantiomers at MOR-Gal_1_R, which could explain their divergent effects on the VTA MOR. First, we evaluated possible differences in binding affinity of (R,S)-MTD, (R)-MTD, and (S)-MTD. We performed radioligand binding experiments in membrane preparations from HEK-293 cells stably transfected with human MOR alone and with human MOR-Gal_1_R^16,37^. The results of competitive inhibition experiments using the MOR antagonist [^3^H]naloxone (1.7 nM) *versus* increasing concentrations of the ligands (**Supplementary Fig. 6**) were analyzed with the ‘dimer receptor model’ (see Methods). In both cell lines and for the three compounds, a significantly better fit was obtained for biphasic versus monophasic curves (p < 0.05 in all cases), indicating the preferred dimeric structure of MOR, forming heteromers or not forming heteromers with Gal_1_R, as previously shown^37^. **Supplementary Table 1** shows that (R,S)-MTD, (R)-MTD and (S)-MTD bind MOR with two different affinities and negative cooperativity, both in MOR and MOR-Gal_1_R cells. None of the obtained binding parameters show significant differences between MOR and MOR-Gal_1_R cells for any of the ligands, indicating that the MOR-Gal_1_R-dependent changes in the pharmacodynamic properties of (S)-MTD are not related to changes in its affinity for the MOR, but likely to its intrinsic efficacy. As expected, (S)-MTD had 14 times lower affinity than (R)-MTD in both cell types.

**Figure 4:**
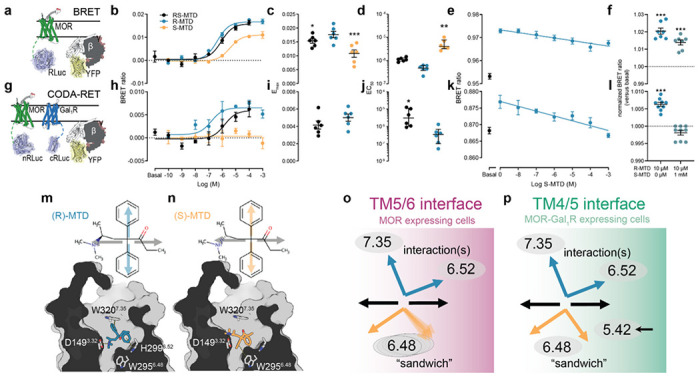
MOR-Gal_1_R heteromer-dependent loss of efficacy of (S)-MTD. **a-f,** BRET experiments in HEK-293T cells cotransfected with MOR fused to RLuc and the α subunit of the Gi protein fused to YFP (schematically shown in **a**), **g-l.** CODA-RET experiments in HEK-293T cells cotransfected with MOR fused to nRLuc, Gal_1_R fused to cRLuc and Gi-YFP (schematically shown in **g**). In **b** and **h**, representative experiments with concentration-responses of (R,S)-MTD (black), (R)-MTD (blue), and (S)-MTD (orange); values represent the mean ± standard error of the mean of triplicates; in **c-d** and **i-j**, corresponding E_max_ and EC_50_ values from 6 independent experiments with triplicates, shown as dots and presented with the mean ± standard error of the mean or median with interquartile ranges, respectively; asterisks are compared to (R)-MTD values. In **e** and **k**, representative experiments of the effect of increasing concentrations of (S)-MTD on BRET and CODA-RET values obtained with (R)-MTD at 100 nM; values represent the mean ± SEM of triplicates; in **f** and **I**, corresponding BRET and CODA-RET values of the effect of (R)-MTD (100 nM) in the presence and absence of (S)-MTD (1 μM) from 7 and 9 independent experiments with triplicates, shown as dots and presented with the mean ± standard error of the mean; asterisks are compared to basal values, **m-n,** Schematic 2D representation of (R)- and (S)-MTD. Grey arrows represent groups of the ligand located toward the conserved protonated amine (left) and toward the -CO-CH2-CH3 moiety (right). The phenyl groups of methadone are depicted by either blue (R-) or orange (S-) arrows. Docking and MD-simulated models (Supplementary Fig. 6) of (R)- and (S)-MTD bound to the MOR. The phenyl rings, in a “V” shaped conformation, point up to interact with H299^6.52^ and W320^7.35^ in (R)-MTD, and point down to interact with W295^6.48^ in (S)-MTD. **o-p**, Previous results^37^ show that MOR forms homodimers via the TM 5/6 interface in the absence of Gal_1_R or via the TM 4/5 interface in the presence of Gal_1_R. MD simulations show that the TM 4/5 triggers an inward movement of TM 5 and, importantly, the inward movement of V238^5.42^ (Supplementary Fig. 8). Thus, in the TM 5/6 interface the phenyl ring of (S)-MTD (flexible arrows) partially restricts the conformation of W295^6.48^ (flexible ellipses), whereas in the TM4/5 interface V238^5.42^ restricts the conformation of the phenyl ring (single arrow) and in consequence W295^6.48^ (single ellipse) in the inactive conformation. BRET = bioluminescence resonance energy transfer; CODA-RET = Complemented donor-acceptor resonance energy transfer; E_max_ = maximal response; EC_50_ = half maximal effective concentration; Gal_1_R = galanin 1 receptor; MD = molecular dynamics; MOR = mu opioid receptor; MTD = methadone. *P < 0.05, **P < 0.01, ***P < 0.001

BRET experiments were performed to evaluate differences in the intrinsic efficacy of (R,S)-MTD, (R)-MTD and (S)-MTD at the MOR (Fig. 4a). MOR-Rluc and Gi-YFP constructs were transiently co-transfected to HEK-293T cells, and concentration-response curves of (R,S)-MTD, (R)-MTD, and (S)-MTD were analyzed for E_max_ and EC_50_ values (Fig. 4b–d). As expected, E_max_ for (S)-MTD was significantly lower than for R-MTD (about 30% lower, Fig. 4c), and EC_50_ for (S)-MTD was significantly higher than for R-MTD (about 10 times; Fig. 4d). Thus, relative to (R)-MTD, (S)-MTD is a partial and less potent MOR agonist.

CODA-RET experiments were then performed to determine whether MOR-Gal_1_R heteromerization might determine the specific pharmacodynamic profile of (S)-MTD (see Methods) (Fig. 4g). HEK-293T cells were co-transfected with MOR fused to nRLuc (MOR-nRLuc), Gal_1_R was fused to cRLuc (Gal_1_R-cRLuc) and Gi-YFP (Fig. 4h–j). In the presence of Gal_1_R, no detectable increase of response (BRET ratio) could be obtained with (S)-MTD, while the dose-response curve of (R,S)-MTD was shifted to the right, with an EC_50_ value significantly higher than for R-MTD (~10-fold; Fig. 4j). These results, therefore, indicate that (S)-MTD, but not (R)-MTD, changes its pharmacological profile and loses its efficacy for the MOR when forming heteromers with Gal_1_R. This implies that the changes in the pharmacological profile of (R,S)-MTD within the MOR-Gal_1_R heteromer, as previously described^16,37^, depend on the modified pharmacodynamic properties of (S)-MTD. Consistent with this, increasing concentrations of (S)-MTD progressively counteracted the effect of a minimal concentration with maximal effect of (R)-MTD (10 μM) (Fig. 4k). At the highest concentration of (S)-MTD (1 mM), the effect of (R)-MTD was completely blocked, and CODA-RET measurements were not significantly different from basal values (Fig. 4I). As a control, the same design was applied with BRET experiments with the MOR alone. In this case, the highest concentration of (S)-MTD (1 mM) did not counteract the effect of (R)-MTD (10 μM) (Fig. 4e, f), and only decreased its effect to the expected maximal level of efficacy of (S)-MTD. These results, therefore, complement those obtained with *in vivo* and *ex vivo* experiments in the VTA (microdialysis and [^35^S]GTP*γ*S) and with locomotor activation and psychomotor sensitization in mice, and provide strong evidence for their mediation by MOR-Gal_1_R heteromers.

### Molecular mechanism of the MOR-Gal_1_R-dependent pharmacodynamic profile of (S)-MTD

The recently reported structure of MOR in complex with fentanyl^38^ can be used as a template to understand the pharmacological differences among the enantiomers of (R,S)-MTD at the molecular level. We first performed five replicas of unbiased 1 μs molecular dynamics (MD) simulations of (S)-MTD and (R)-MTD docked into the MOR monomer (see Methods). Root-mean-square deviations (rmsd) of the simulations show that the proposed docking models of (S)-MTD and (R)-MTD remained highly stable (**Supplementary Fig. 7**). In these models, the protonated amine of (S)-MTD and (R)-MTD forms the conserved ionic interaction with D149^3.32^, and both phenyl groups adopt a “V” shaped conformation in the orthosteric binding site but, importantly, with significant differences (Fig. 4m, n). In (R)-MTD, both phenyl rings point up to form T-shaped aromatic interactions with H299^6.52^ and W320^7.35^, whereas the phenyl rings of (S)-MTD point down to interact with W295^6.48^ in a “sandwich” mode in which the aromatic Trp ring is between both phenyl rings (**Supplementary Fig. 7** shows a detailed analysis of the binding modes). We suggest that the phenyl ring of (S)-MTD positioned between W295^6.48^ and TM 5, absent in (R)-MTD, restricts the necessary movement of W295^6.48^ for activation^38,39^, which explains the decreased ability of (S)-MTD to activate MOR.

To understand the inability of (S)-MTD to activate MOR in the presence of Gal_1_R, at the molecular level, we first needed to computationally model the MOR-Gal_1_R heteromer (**Supplementary Fig. 8**). Previously reported bimolecular fluorescence complementation (BiFC) and total internal reflection fluorescence (TIRF) microscopy experiments, in the presence of synthetic peptides corresponding to different TM domains of MOR and Gal_1_R, revealed that the interface for the MOR-MOR homodimer changed from the TM 5/6 to the TM 4/5 interface in the absence and presence, respectively, of Gal_1_R^37^. Thus, we hypothesized that the MOR-MOR homodimer interacting via the TM 4/5 interface disables (S)-MTD to activate MOR. To test this hypothesis, we performed five replicas of unbiased 1 μs MD simulations of the MOR-MOR homodimer, constructed via both the TM 5/6 (not interacting with Gal_1_R) and TM 4/5 (interacting with Gal_1_R) interfaces, in complex with Gi (see Methods and **Supplementary Fig. 8**). These simulations showed that, in contrast to the TM 5/6 interface, TM 5 of the active Gi-bound protomer moved the extracellular part of TM 5 inward in the TM 4/5 interface. Importantly, this movement of TM 5 relocated the position of the key V238^5.42^ (**Supplementary Fig. 8**). Fig. 4o, p summarizes these findings. In the TM 5/6 interface (Fig. 4o), W295^6.48^ is only partially restricted (depicted as flexible ellipses) by the phenyl ring of (S)-MTD because the dynamic behavior of the ligand is not fully constrained by the partner protomer (depicted as flexible arrows). In contrast, in the TM 4/5 interface (Fig. 4p), the inward movement of V238^5.42^ fully constrained (depicted as a single arrow) the phenyl ring of (S)-MTD, maintaining W295^6.48^ in the inactive conformation (depicted as a single ellipse).

## Discussion

(R,S)-MTD is a DEA Schedule II controlled medication with known abuse liability that is prescribed for pain management and treatment of OUD. However, the individual contributions of its enantiomers to its abuse liability and clinical efficacy are not well understood. We found that both (R)-MTD and (S)-MTD produced full agonistic effects on analgesia but only (R)-MTD was reliably self-administered. These findings are in agreement with results from recent studies indicating that (S)-MTD does not lead to reinforcing effects, physical dependence nor withdrawal signs in rats^40^ and that it lacks opioid effects, or withdrawal signs and symptoms in humans^5^, suggesting that the abuse liability of (R,S)-MTD is mediated by (R)-MTD and not by (S)-MTD. Indeed, our data indicate that (S)-MTD can attenuate the abuse liability of (R)-MTD under some conditions.

Although some experimental and clinical effects of (S)-MTD have been attributed to its NMDAR antagonism^5,7–9,41^, we demonstrate here that, at pharmacologically significant doses, (S)-MTD does not interact with NMDARs *in vivo*. Instead, (S)-MTD significantly occupies MORs at doses that promote the classical behavioral effects of opioids in rats: analgesia, catalepsy, and hypothermia. For example, the effective dose at which (S)-MTD produced analgesia is within the range of doses used to produce antidepressant-like effects in rats^6,7^. For both (R)-MTD and (S)-MTD, the predicted free brain concentrations coincided with their *in vitro* MOR affinities as well as their capacity to selectively occupy MORs *in vivo*. In contrast, we failed to detect *in vivo* NMDAR occupancy at the same doses where MOR occupancy was observed. Therefore, we can conclude that (S)-MTD selectively binds MORs at brain concentrations relevant to its analgesic and antidepressant-like efficacy. Thus, the currently assumed role of NMDAR blockade in the purported antidepressant effects of (S)-MTD should be reassessed and explained in the frame of its MOR agonistic properties.

We demonstrated that (S)-MTD does not promote activation of the dopaminergic system, likely due to the inability of (S)-MTD to activate MOR-Gal_1_R in the VTA, previously shown to mediate the dopaminergic effects of opioids^16^. On the other hand, (R)-MTD promoted a significantly stronger activation of the VTA dopaminergic system than the reported effect of (R,S)-MTD^16^. The specific lack of effect of (S)-MTD was due to its loss of intrinsic efficacy for MOR-Gal_1_R, which also explains its antagonism of (R)-MTD-induced effects in the VTA including dopamine release, [^35^S]GTP*γ*S recruitment, and locomotor activation.

The significant analgesic and cataleptic effects of (S)-MTD indicate dopamine-independent mechanisms, not mediated by MOR-Gal_1_R. As opposed to neuroleptic-induced catalepsy, opioids do not induce catalepsy by inhibiting striatal dopaminergic neurotransmission, but possibly by inhibiting the MOR-expressing striatal and pallidal GABAergic neurons that project to the output structures of the basal ganglia^30,42,43^. These two functionally opposite MOR-dependent effects, locomotor activation and catalepsy, are both present but differentially dominate in mice and rats, respectively. In fact, locomotor activation can also be elicited in rats with the intracranial injection of opioids in the VTA^44^, and catalepsy has been reported with relatively high doses of opioids in mice^45^ and likely contributes to the descending limb of the dose response curve for opioid-induced locomotor activity in mice^26,28^.

The question of whether dopamine neurotransmission underlies the reinforcing properties of opioids has been a matter of debate^46–48^. Nevertheless, results from chemogenetic and optogenetic experiments in mice^49^ strongly support the involvement of VTA dopamine neurons projecting to the ventral striatum in driving heroin reinforcement. The present results showing IVSA of (R,S)-MTD and (R)-MTD but not (S)-MTD, are consistent with the dopaminergic hypothesis. Nevertheless, at high doses, (S)-MTD was able to substitute for (R)-MTD in rats trained on (R)-MTD, which support the involvement of additional non-dopaminergic mechanisms in opioid reinforcement. Importantly, the dose response of (R,S)-MTD IVSA was qualitatively different from that of (R)-MTD, with a significantly higher peak and a pronounced shift to the right. This could be explained by (S)-MTD counteracting the effect of (R)-MTD at sufficiently high doses of (R,S)-MTD.

One potential adverse effect of (R,S)-MTD use is that it can cause cardiac arrythmia^50^. This has been attributed to high concentrations of the drug and perhaps to the presence of (S)-MTD, which one study reported blocks the Ether-à-go-go-Related Gene 1 (hERG) channel 3.5-fold more potently than (R)-MTD^51^. However, the stereoselective contribution of (R,S)-MTD enantiomers to these effects has been challenged^52^. We did not observe any meaningful binding for (R)-MTD and (S)-MTD at hERG, even at a concentration of 10 μM. Furthermore, recent clinical studies assessing (S)-MTD for depression have not reported any cardiac arrythmias^9^. It is therefore unclear to what extent (S)-MTD contributes to the cardiac effects of (R,S)-MTD.

Combined, the present results help explain the clinical profile of (R,S)-MTD, since they indicate that its lower abuse liability, as compared with other opioids^16,53^, are due to the specific effect of (S)-MTD at MOR-Gal_1_R heteromers which counteract rewarding and dopamine releasing effects of (R)-MTD. In addition, the results suggest that the separation of reinforcing vs. therapeutic effects of (R,S)-MTD^16^ should be significantly augmented with (S)-MTD. (S)-MTD could then be used clinically as analgesic, antidepressant, or for the treatment of opioid withdrawal or restless legs syndrome, with the anticipation of low abuse liability. The results of the *in silico* analysis provide information about the possible molecular mechanism underlying MOR-Gal_1_R heteromer-dependent pharmacodynamic properties of (S)-MTD, which could also guide the search for novel (S)-MTD-like therapeutics.
